# Consciousness isn’t “hard”—it’s human psychology that makes it so!

**DOI:** 10.1093/nc/niae016

**Published:** 2024-04-03

**Authors:** Iris Berent

**Affiliations:** Department of Psychology, Northeastern University, 360 Huntington Ave., Boston, MA 02115, USA

**Keywords:** consciousness, “the hard problem of consciousness”, intuitive psychology, Dualism, Essentialism

## Abstract

Consciousness arguably presents a “hard problem” for scholars. An influential position asserts that the “problem” is rooted in ontology—it arises because consciousness “is” distinct from the physical. “Problem intuitions” are routinely taken as evidence for this view. In so doing, it is assumed that (i) people do not consider consciousness as physical and (ii) their intuitions faithfully reflect what exists (or else, intuitions would not constitute evidence). New experimental results challenge both claims. First, in some scenarios, people demonstrably view consciousness as a physical affair that registers in the body (brain). Second, “problem intuitions” are linked to psychological biases, so they cannot be trusted to reflect what consciousness is. I conclude that the roots of the “hard problem” are partly psychological. Accordingly, its resolution requires careful characterization of the psychological mechanisms that engender “problem intuitions.”

## Why consciousness is “hard”: ontology or psychology?

Consciousness arguably presents a “hard problem” for scholars (Chalmers 1996). The challenge is to explain how phenomenal experience emerges from the physical.

An influential position asserts that the “problem” arises because consciousness *is* distinct from the physical; at its core, then the “problem” is ontological (hereafter, “ontological Dualism”) (Chalmers 1996). In line with this position, people sometimes intuit that consciousness can dissociate from physical properties (e.g. in zombies—creatures that maintain one’s body but not one’s consciousness), just as the ontological approach suggests.

But, alas, human intuitions are hardly infallible, as people fall prey to a wide array of psychological illusions. “Problem intuitions,” then, do not necessarily reflect what consciousness *is*. Rather, they could merely indicate how consciousness *seems* through the prism of our internal psychological biases.

Indeed, several philosophers have long argued that consciousness is an illusion (Dennett 1991, Humphrey 2011, Frankish 2016). Consciousness, in this view, is not available to us immediately (as the “Realists” claim (Chalmers 1996)); perhaps, it does not even exist (Frankish 2016). But while “Illusionists” and “Realists” debate *why* we view consciousness as distinct from the physical, both sides agree that we *do*. Indeed, it is precisely *because* we (laypeople and scholars) presumably *perceive* consciousness as not physical that the ontological question arises in the first place: if consciousness *seems* not physical, then perhaps it *is* so.

This assumption, however, is uncertain. Many “problem intuitions” do not explicitly gauge the perceived physicality of conscious states, and the possibility that, in some scenarios (e.g. the zombie case), consciousness seems ethereal does not prove that it always seems so.

A recent experimental investigation (Berent 2023a) has directly gauged people’s intuitions, and the results were surprising. In one famous scenario (“Mary in the black and white room”), people actually intuited that gaining a conscious experience is a *physical* affair that distinctly registers in the body (in the brain).

These results strongly challenge the ontological Realist view (Chalmers 1996). If consciousness sometimes seems ethereal (for Zombies) and sometimes physical (for “Mary’s” case), then surely, these intuitions cannot reflect what consciousness *is*. As I next show, all these intuitions—physical or not—can be traced to well-known psychological biases. Accordingly, the roots of the “hard problem” are partly psychological.

Here, I describe the experimental results and introduce their psychological origins. While this commentary relies on the previous experimental report (Berent 2023a), its conclusions extend it in several ways. First, this commentary explicitly contrasts laypeople’s intuitions in two problems—those of “Mary” and Zombies. Second, it considers the implications of the empirical results to the philosophical questions of Illusionism and the meta-problem. Finally, and most critically, by focusing on the theoretical problem at hand (free from the burden of experimental details), this discussion seeks to highlight the broader theoretical considerations and bring them to the forefront.

## Intuitive vs. ontological Dualism

As noted, the ontological account asserts that phenomenal experience *is* distinct from the physical. The psychological account, by contrast, attributes “problem intuitions” to tacit psychological biases that can render consciousness *seem* that way.

Psychological biases are implicit reasoning heuristics that shape how people tacitly construe their psychological reality. One such bias is intuitive Dualism—the tacit belief that the mind is ethereal, distinct from the physical body (Stanovich 1989, Bloom 2004, Berent 2020, Barlev and Shtulman 2021).

Unlike the philosophical doctrine of Dualism (an explicitly account of what exists, ontologically), intuitive Dualism is a psychological bias that guides how laypeople reason about bodies and minds *implicitly*—people are often unaware that they segregate bodies and minds. Indeed, intuitive Dualism has been shown to affect reasoning in adults and young children (Bering and Bjorklund 2004, Hood et al. 2012, Chudek et al. 2018), in both western cultures (Stanovich 1989, Bloom 1994, Bering et al. 2005, Cohen and Barrett 2008, Slingerland and Chudek 2011, Forstmann et al. 2012, Forstmann and Burgmer 2015, Heflick et al. 2015, Lane et al. 2016, Weisman et al. 2017, Berent 2021, Berent et al. 2021, Sandoboe and Berent 2021, Berent and Platt 2021a, 2021b) and small-scale societies (Astuti and Harris 2008, Cohen et al. 2011, Watson‐Jones et al. 2017, Boyer 2018, Chudek et al. 2018, Weisman et al. 2021). Furthermore, past research has linked intuitive Dualism to early systems of core knowledge (Bloom 2004), suggesting that it emerges in humans naturally and spontaneously (Berent et al. 2022, Berent 2023b). To distinguish this tacit psychological bias from the philosophical doctrine by the same name, I refer to it as “intuitive.”

Intuitive Dualism presents a ready explanation for why consciousness seems distinct from the physical. Since consciousness is a mental state, and the mind seems ethereal, then, *by default*, people ought to view consciousness as nonphysical for reasons that are entirely psychological.

Nonetheless, psychological biases (such as intuitive Dualism) may not invariably render the mind (consciousness included) ethereal. Afterlife beliefs nicely illustrate these contradictory psychological intuitions.

Afterlife beliefs are common across cultures (Boyer 2018). People maintain that disembodied agents persist after death, despite the demise of their bodies (Bering and Bjorklund 2004, Berent et al. 2022)—this is exactly what is expected by intuitive Dualism. And yet, these afterlife creatures also seem partly embodied: in need of food, drink, and physical artifacts (Lomnitz-Adler 2005, Barlev and Shtulman 2021). How can afterlife creatures seem both ethereal and embodied?

This tension can be readily explained by the hypothesis that intuitive Dualism is a “soft” violable constraint that interacts with conflicting cognitive constraints (Berent 2023c). While intuitive Dualism mandates that the mind is ethereal (e.g. persistent without the body), another psychological bias (intuitive Essentialism) might require agents to possess bodily properties. Constraint interaction (detailed below), then, could explain why afterlife creatures do not seem purely ethereal but partly embodied.

Several conclusions follow. First, intuitions about psychological traits, consciousness included, could arise from psychology (intuitive Dualism), rather than from ontology (ontological Dualism). Second, since intuitive Dualism is soft and violable, its effects can be context dependent: in one scenario, participants could view consciousness as distinct from the physical, but in others (which draw attention to the body) consciousness might seem chimeric, or even as squarely physical. Thus, the fact that consciousness seems disembodied in one situation offers no guarantee that these intuitions generalize to others.

I now turn to demonstrate these conflicting facets of intuitive Dualism in two representative “problem intuitions”—“zombies” and “Mary in the black and white room.” “Zombies” suggest that consciousness is ethereal, distinct from the physical; but in the case of “Mary,” people intuit just the opposite—that consciousness is physical.

## Zombie intuitions

Zombies—creatures that maintain one’s body but are devoid of consciousness—have been cited as evidence that consciousness can dissociate from the physical; this is precisely what the ontological account predicts (Chalmers 1996).

**Figure 1.**
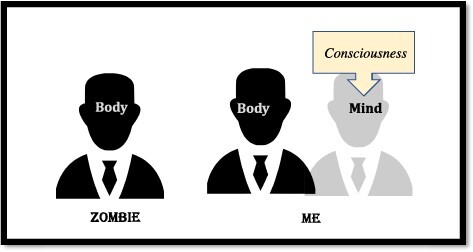
The psychological account of “zombie” intuitions (reproduced from Berent (2023a). The “hard problem of consciousness” arises from human psychology. “Open Mind: Discoveries in Cognitive Science”) (Berent 2023a)

But it is also easy to see how intuitive Dualism can capture this case (as well as other dissociations of consciousness from the physical, e.g. color spectrum). As noted, intuitive Dualism renders the mind distinct from the body, and consciousness is a mental state. Accordingly, by default, intuitive Dualism ought to render consciousness ethereal, so consciousness and the physical body ought to dissociate: I am consciousness and body; but my zombie could consist of body only (Fig. 1).

Admittedly, the force of this case is limited. All it shows is that some critical facts about consciousness can be captured in *either* ontological *or* psychological terms. What we have, then, is a stalemate between two competing explanations, not a psychological victory.

Other scenarios, however, could conceivably shift one’s intuitions about the physical embodiment of consciousness, and that shift could help break the tie between the ontological and psychological perspectives.

From the ontological perspective, such shifts are puzzling. If physicalist intuitions reflect what consciousness is, and consciousness is invariant, then so must be our intuitions. The psychological account, by contrast, predicts that “intuitive Dualism” is violable, hence, context dependent. Thus, if the problem framing were to focus attention on an agent’s body, and concerns with intuitive Dualism were downplayed, then consciousness might seem not ethereal but physical—a bodily affair. “Mary in the black and white room” (Jackson 1982, Chalmers 1996) elicits these very intuitions, so let’s review it next.

## Mary in the black and white room

Suppose Mary is an expert on the neuroscience of color vision. Mary knows everything there, is to know about how color vision works, and how it is encoded in the brain. Yet Mary herself has never seen color, as she lives in a black and white room. Now, suppose Mary were to step out of the black and white room and see a red rose for the first time. How would this experience change her grasp of color vision—would she gain something new?

Many readers would claim that indeed, Mary would gain something new and significant. We now consider the two competing explanations for this intuition.

### The ontological account

In the ontological view, Mary’s (new) phenomenal experience is significant because it goes beyond her (previous) knowledge of the physical facts. Presumably, this is so because consciousness is distinct from the physical (Chalmers 1996)—either unrelated to physical brain processes (hereafter: “not physical”) or opposite to them (hereafter: nonphysical).

What intuitions are expected, under this proposal? The “ontological account” does not fully address this question, as its concern is with the “hard problem,” not with the psychological mechanism that yields problem intuitions (the “meta-problem”). To bridge the gap between the two problems, I suggest several “linking hypotheses.”

First, since the ontological account takes “problem intuitions” as evidence for ontology, it follows that people ought to be largely privy to what exists (or else, their intuitions could not have possibly provided reliable evidence regarding ontology). In particular, “problem intuitions” arise from the perceived gap between Mary’s phenomenal experience and the physical. Thus, people ought to intuit that Mary’s phenomenal experience is distinct from the physical—either not physical or nonphysical, but they certainly *shouldn’t* view it as physical.

Second, intuitions about the physicality of Mary’s phenomenal experience ought to be linked to intuitions regarding its significance. Thus, if Mary’s phenomenal experience is nonphysical, then the two intuitions (of physicality and “significance”) ought to correlate negatively—the more significant the subjective experience, the less physical it ought to seem; if Mary’s experience is merely not physical, then no correlation is expected. Certainly, one *shouldn’t*expect a *positive* correlation—that the more significant is the experience, the more physical it seems.

Finally, to gauge verbal intuitions regarding Mary’s conscious experience, one ought to describe its significance using some adjective; in the present experiments (Berent 2023a), participants were asked to judge whether Mary’s new experience is “transformative.”

### The psychological account

The psychological account sharply differs from the ontological position on two key questions: (i) what Mary has gained and (ii) why it’s significant.

Consider first what Mary has gained. In her “before” state, while in the black and white room, Mary had only knowledge (of color vision). “After” she has stepped out of the black and white room and saw the red rose, she has gained perceptual experience (rather than new knowledge, as the ontological account suggests).

Critically, in the context of Mary’s example, that subjective perceptual experience may not necessarily be nonphysical (*contrary* to what we have just suggested in the zombie’s case). This is so because (by hypothesis) embodiment intuitions are context-sensitive, and the context of Mary’s case differs markedly from the previous zombie example.

The zombie case invites us to consider whether consciousness dissociates from body. The problem framing, then, contrasts consciousness with the physical body (e.g. arms and legs), and, when compared to the body, the mind (consciousness included) seems ethereal (Bering and Bjorklund 2004).

**Figure 2.**
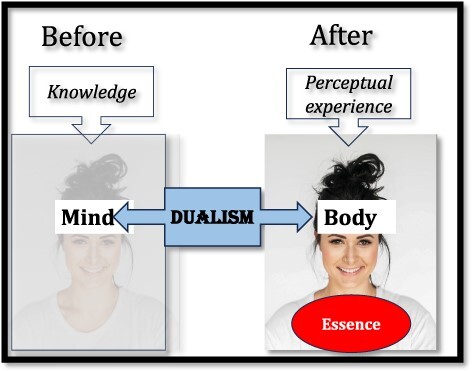
The psychological account of intuitions concerning Mary’s case (reproduced from Berent (2023a). The “hard problem of consciousness” arises from human psychology. “Open Mind: Discoveries in Cognitive Science”) (Berent 2023a)

Mary’s case, by contrast, compares Mary’s consciousness to her knowledge, and intuitively, knowledge seems quintessentially ethereal (Berent et al. 2021, 2022, Berent 2023b)—people do not spontaneously anchor knowledge in the brain (Sandoboe and Berent 2021). Against that ethereal backdrop, “seeing color” would seem relatively embodied—it is with our eyes, after all, that we see. So, in the psychological account, what Mary has gained is *perceptual experience*, and that experience is firmly *embodied* (Fig. 2).

Why is this new experience so significant? The ontological account would lead us to suggest that’s so because consciousness *is* distinct from the physical. But in the psychological account, the significance of Mary’s experience arises from an entirely different source.

Mary’s case invites the reader to evaluate a *change* incurred by her new experience. And past research has shown that, to determine whether a change to an agent is significant (e.g. does painting a racoon turn it into a skunk? (Keil 1986)), people evaluate the effect of the change on one’s essence (Keil 1986, Gelman 2003). Changes that tap into- or alter the hidden essence are significant; those that don’t (e.g. a coat of paint) aren’t. And since our essence seems to lie within the body (Berent, 2021), only changes that pertain to one’s body can be transformative.

Returning to Mary’s case: seeing—Mary’s new experience—seems embodied, more so than her previous knowledge of color, which, by comparison, is ethereal. So, in the psychological account, Mary’s color experience is transformative precisely because it is embodied.

### Competing predictions

Summarizing, the ontological and psychological accounts are quite clear, and their predictions for Mary’s case are diametrically opposed. In the ontological account, Mary’s phenomenal experience consists of new knowledge; the psychological account views it as new perception. The ontological account further states that what Mary has gained is significant because it is *distinct* from the physical—either simply not physical or squarely nonphysical (i.e. disembodied); the psychological account states that the significance of the experience arises precisely “because” it is physically embodied; in fact, it explicitly predicts that Mary’s subjective experience can be significant *only* if it seems embodied. Furthermore, intuitions about the significance and physicality of Mary’s experience ought to correlate positively; this contrast with the null/negative correlations, expected by the ontological alternative.

### An experimental adjudication

Armed with these precise, conflicting predictions, we can now evaluate the ontological and psychological explanations experimentally. A recent series of experiments has done just that (Berent 2023a).

One experiment evaluated which kind of new subjective experiences seem transformative—perceptions or knowledge, and why—do transformative subjective experiences seem embodied or disembodied?

**Figure 3.**
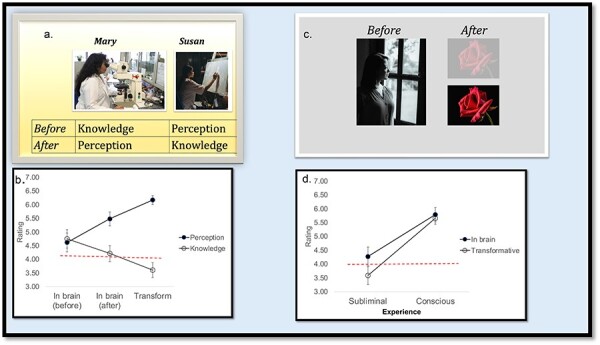
Experimental manipulations and results. One experiment (a) contrasted the “before” and “after” states of Mary (the neuroscientist) and Susan (the artist); results (b) showed that Mary’s conscious perception was considered both transformative and likely to “show up in her brain,” whereas Susan’s conscious knowledge was not (*n* = 57 participants). In a second experiment, Mary’s first encounter with the red rose (“after” leaving the black and white room) was either subliminal or conscious (c); results (d) showed that the conscious experience was considered transformative and likely to show up in the brain, whereas her subliminal experience was not (*n* = 29 participants). In both experiments, ratings were given on a 1–7 scale; the scale’s “neutral” midpoint is depicted by the dotted line. Errors bars are SE. Data are reproduced from Berent (2023a). The “hard problem of consciousness” arises from human psychology. “Open Mind: Discoveries in Cognitive Science” (Berent 2023a)

To find out, participants were presented with two subjective experiences (Fig. 3a)—that of Mary (the neuroscience expert who sees color for the first time) and Susan—a renowned artist with exceptional tacit understanding of “how to make color speak” but no explicit knowledge of the relevant science. Thanks to a crash course in science, Susan now gains that subjective knowledge for the first time. Participants were invited to evaluate how transformative are these two experiences and how embodied they are (i.e. will they “show up” in a brain scan?).

What do participants think, then? Do they believe transformative experiences are embodied or disembodied? And are “transformation” and “embodiment” intuitions linked negatively (as expected by the ontological account) or positively (as the psychological account predicts)? Finally, do participants believe that both conscious experiences (Mary’s and Susan’s) are transformative or is it only the case for Mary’s?

The results (Berent 2023a) came back loud and clear (Fig. 3b). Yes, people do consider Mary’s perceptual experience transformative, and yes, the sense of transformation and embodiment are linked. But, contrary to the ontological account, Mary’s subjective experience (“after”) seemed *more* strongly embodied than her previous knowledge (rather than less so), and the sense of embodiment and transformation correlated *positively*, not negatively. Finally, unlike Mary’s perceptual experience, Susan’s conscious knowledge seemed neither transformative nor embodied (in the brain). This is precisely what the psychological account predicts.

Another experiment (Berent 2023a) clarified that people indeed believe that, when Mary gains conscious experience, that experience is etched in her body. Here, participants were invited to imagine that Mary (who had previously lived in the black and white room) were to see color for the first time. In one condition, that new experience (“after”) happened subliminally, for a fraction of a second, so Mary is not conscious of what she saw, even though the percept is demonstrably registered (e.g. Mary can subsequently recognize the word “rose” more readily). In another condition, the red rose was fully visible, such that Mary is now consciously aware of what she saw (Fig. 3c). The two conditions—the subliminal and conscious—thus differ minimally, only on whether they produce subjective experience.

When asked to evaluate the effect of the conscious and subliminal conditions, participants considered only the conscious experience as transformative (and not the subliminal percept)—this is only expected. Remarkably, participants also considered the conscious experience as more strongly embodied (Fig. 3d). And, as in the previous study, the sense of embodiment and transformation correlated *positively*: the more embodied was Mary’s conscious experience, the more transformative it seemed.

A third experiment evaluated the link between consciousness intuitions and psychological Dualism. Results confirmed that participants indeed veer toward Dualism (e.g. they believe that thoughts are generally less likely to form part of the body than percepts). Critically, the stronger the Dualist intuitions, the more likely were participants to consider Mary’s conscious perception transformative.

The results make it clear that, in participants’ view, a transformative conscious experience isn’t distinct from the physical—as the ontological account ought to predict. Rather, it is physically embodied, as expected by the psychological explanation.

## Concluding remarks

In this commentary, I outlined a psychological explanation for the “hard problem” and contrasted it with the “ontological alternative.” The experimental results clearly favored the psychological account.

In particular, several findings directly challenge the ontological position. First, people consider Mary’s phenomenal experience as physical rather than as nonphysical (or not physical, as the ontological account suggests). Second, the significance of this experience is positively linked to its perceived physicality (rather than negatively, or unrelated, as expected by the ontological view). Third, “problem intuitions” bear the signatures of intuitive Dualism.

These findings suggest that consciousness intuitions are constructed by psychological biases. As Nicholas Humphrey puts it, consciousness is a “self-generated show” (Humphrey 2011, p. 50). The experimental results align with the philosophical position of Illusionism (Dennett 1991, Humphrey 2011, Frankish 2016) and challenges Chalmers’ claim (Chalmers 1996) that consciousness intuitions are immediately available to us and they speak to what exists (i.e. Realism).

The present results, however, address the Illusionist–Realist debate only partially. Indeed, in the Strong Illusionist view (Frankish 2016), it is not merely our intuitions about consciousness that are faulty—consciousness itself is an illusion. The present results cannot evaluate this strong claim. Since the experiments only concern laypeople’s intuitions about consciousness, they cannot speak to what consciousness *is*. Still, the results strongly suggest that consciousness intuitions are authored by the mind, so they cannot be trusted to reflect ontology. At its core, then, the “hard problem” likely arises from psychology.

These conclusions by no means deny that a “hard problem” exists. In fact, the experimental results make it clear that participants are exquisitely tuned to the anchoring of consciousness in the physical body. For laypeople, then, the “hard problem” is very much an urgent psychological problem. But the psychological “hard problem” is quite distinct from the one outlined by philosophers (Chalmers 1996). First, the psychological “hard problem” is tacit: it arises from the tension between two intuitive psychological biases—from intuitive Dualism and Essentialism, not from explicit philosophical deliberations about what exists (ontologically). Furthermore, laypeople’s solution to the “problem” does not necessarily view consciousness as distinct from the physical (contrary to the ontological position) (Chalmers 1996).

I should note that the present results from western participants are limited, insofar that they do not evaluate the universality of consciousness intuitions across cultures. My goal here, however, was not to assess the scope of consciousness intuitions but their putative origins. Since discussions of the “hard problem” have been informed by untested assumptions about consciousness intuitions, and since these assumptions were made by western philosophers, to evaluate those assumptions, I chose to first examine western participants. As noted, these assumptions were not borne out. Future research ought to extend this inquiry across cultures.

The conclusion that consciousness intuitions are constructed psychologically further underscores the intimate link between the “hard problem” and the problem of explicating the psychological mechanisms that give rise to intuitions (the “meta-problem” (Chalmers 2018)). Neglecting the “meta-problem” can not only obscure the “hard problem” but further confound one’s command of the empirical facts.

Embodiment intuitions illustrate this danger. It is tempting to conclude that if zombies are conceivable, then consciousness must invariably seem distinct from the physical (possibly, because it is so). But as one considers the violable constraints that govern psychological intuitions, it quickly becomes evident that what’s intuitively true about zombies may not necessarily hold for Mary, as the results indeed suggest. Thus, the investigation of the “hard problem” and “meta-problem” are interdependent (Chalmers 2018). Psychological analysis, such as the one outlined here, can contribute to philosophical inquiry.
